# Recent Advances in Oligonucleotide Therapeutics in Oncology

**DOI:** 10.3390/ijms22073295

**Published:** 2021-03-24

**Authors:** Haoyu Xiong, Rakesh N. Veedu, Sarah D. Diermeier

**Affiliations:** 1 Department of Biochemistry, University of Otago, Dunedin 9016, New Zealand; haoyu.xiong@otago.ac.nz; 2 Centre for Molecular Medicine and Innovative Therapeutics, Murdoch University, Perth 6150, Australia; R.Veedu@murdoch.edu.au; 3 Perron Institute for Neurological and Translational Science, Perth 6009, Australia

**Keywords:** antisense oligonucleotides, siRNA, aptamers, DNAzymes, cancers

## Abstract

Cancer is one of the leading causes of death worldwide. Conventional therapies, including surgery, radiation, and chemotherapy have achieved increased survival rates for many types of cancer over the past decades. However, cancer recurrence and/or metastasis to distant organs remain major challenges, resulting in a large, unmet clinical need. Oligonucleotide therapeutics, which include antisense oligonucleotides, small interfering RNAs, and aptamers, show promising clinical outcomes for disease indications such as Duchenne muscular dystrophy, familial amyloid neuropathies, and macular degeneration. While no approved oligonucleotide drug currently exists for any type of cancer, results obtained in preclinical studies and clinical trials are encouraging. Here, we provide an overview of recent developments in the field of oligonucleotide therapeutics in oncology, review current clinical trials, and discuss associated challenges.

## 1. Introduction

According to the Global Cancer Statistics 2018, there were more than 18 million new cancer cases and 9.6 million deaths caused by cancer in 2018 [1]. Cancer incidence and mortality are growing rapidly throughout the world [1]. While lung, breast, prostate, and colorectal cancers show the highest incidence rates (11.6%, 11.6%, 7.1%, and 6.1%, respectively), lung, colorectal, stomach, and liver cancers are the deadliest cancers (18.4%, 9.2%, 8.2%, and 8.2%, respectively) [1]. The direct cost of cancer was estimated to be $1.16 trillion (USD) globally in 2010 [2]. According to US statistics, the cost for cancer was around 125 billion (USD) in 2010 and is estimated to rise to 158 billion (USD) in 2020 [3]. Conventional treatments for cancer, such as surgery to physically remove cancer tissues and metastases, radiation therapy to damage cancer cells under high energy waves, and chemotherapy therapy utilizing systemic cytotoxic drugs, have significantly improved cancer survival rates. However, the current standard of care for many types of cancer fails to address cancer recurrence and/or metastasis [4,5]. Moreover, conventional therapies are often accompanied by serious adverse effects such as systemic toxicity and multiple drug resistance [6,7]. 

Recent research into immune checkpoints and intrinsic mechanisms of cancer growth resulted in the development of targeted therapies including small molecule inhibitors and monoclonal antibodies as well as cell and immunotherapies, including checkpoint inhibitors and chimeric antigen receptor (CAR) T-cell therapy [7]. Checkpoint inhibitors such as PD-1 and PD-L1 demonstrate significant improvements in the clinic in some types of cancer, but their benefits are limited by fast post-therapy resistance and intrinsic absence of tumor neoantigens to enable blockade, rendering them ineffective for many patients [8,9]. CAR T-cell therapy requires functional tumor-specific antigens on the cell surface of cancer cells to enable targeting, and lack of these challenge the therapeutic purpose of CAR T-cell therapy, especially in solid tumors, which often do not express such antigens and show a 9% overall response rate [10,11,12]. Targeted therapy aims to inhibit cancer cell growth by blocking molecular targets essential for cell growth and tumorigenesis [13]. Many targeted drugs such as anti-EGFR agents and anti-VEGF/VEGFR agents have been approved for cancer treatments, but limitations include rapidly developing resistance and serious adverse effects [14,15]. While both conventional and newly developed treatments have significantly improved cancer survival rates, cancer recurrence and drug resistance remain challenging and indicate the urgent need for new therapeutic strategies.

Oligonucleotide therapeutics are an emerging drug modality, which consists of modified or unmodified short nucleic acid molecules, and includes antisense oligonucleotides (ASOs), small interfering RNA (siRNAs), microRNA (miRNAs), aptamers, and DNAzymes [16]. The mechanism of action of oligonucleotide therapeutics mainly relies on Watson–Crick base pairing to targeted mRNAs, resulting in either gene silencing, a steric block, or altered splicing patterns, with the exception of aptamers, which recognize their targets by their three-dimensional structures [16,17,18,19]. Currently, 13 oligonucleotide therapeutics have been granted new drug approval (NDA) by the U.S. Food and Drug Administration (FDA) for several different indications (Table 1) [20]. The underlying molecular mechanism is similar for all of these indications: a single gene mutation is responsible for these diseases. The approved oligonucleotide therapeutics act via a variety of different mechanisms correcting the affected gene, such as by blocking translation (fomivirsen, the first approved oligonucleotide drug but withdrawn due to safety issues), RNase H dependent degradation (mipomirsen, inotersen and volanesorsen), splicing modulation (eteplirsen, nusinersen, golodirsen, milasen, and casimersen), siRNA dependent degradation (patisiran and givosiran), aptamer binding and blocking (pegaptanib), and aptamer binding and activating (defibrotide) (Table 1) [20]. Defibrotide is different from other approved therapeutics as it consists of a mixture of single-stranded and double-stranded phosphodiester oligonucleotides, which act as aptamers to activate adenosine receptor and plasma membranes of vascular endothelial cells [21].

**Figure 1:**
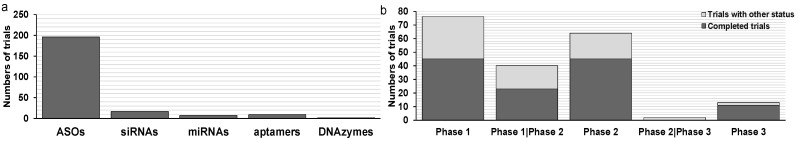
Summary of clinical trials for oligonucleotide therapeutics in oncology. (**a**) Numbers of trials for different types of oligonucleotide therapeutics; (**b**) Trials status. Number of trials were retrieved from Supplementary Materials.

Oligonucleotide therapeutics have been investigated as cancer treatments for decades with promising in vitro outcomes and many have been tested in clinical trials [22]. However, there are currently no approved oligonucleotide therapeutics in oncology yet. Three ASO drugs were granted orphan drug designation (oblimersen for chronic lymphocytic leukemia, PNT2258 for diffuse large B-cell lymphoma, and cobomarsen for mycosis fungoides type cutaneous T-Cell lymphoma). Most oligonucleotide therapeutics for cancer are still in clinical phase 1 or 2. Currently (as of 01/01/2021), a total of 229 clinical trials investigating 60 oligonucleotide drugs in oncology are registered on ClinicalTrials.gov. Of these, 195 are using ASOs as intervention, 17 trials for siRNAs, seven trials for miRNAs, nine trials for aptamers, and one trial for DNAzymes (Figure 1a; Table 2). There are currently 15 oligonucleotide therapeutics in phase 2/3 or phase 3 trials (all for ASOs) (Figure 1b). In this review, we summarize current clinical trials of oligonucleotide therapeutics for cancer. We further discuss potential reasons for previous failures, new developments in the field, and future directions.

## 2. Types of Oligonucleotide Therapeutics

**Figure 2:**
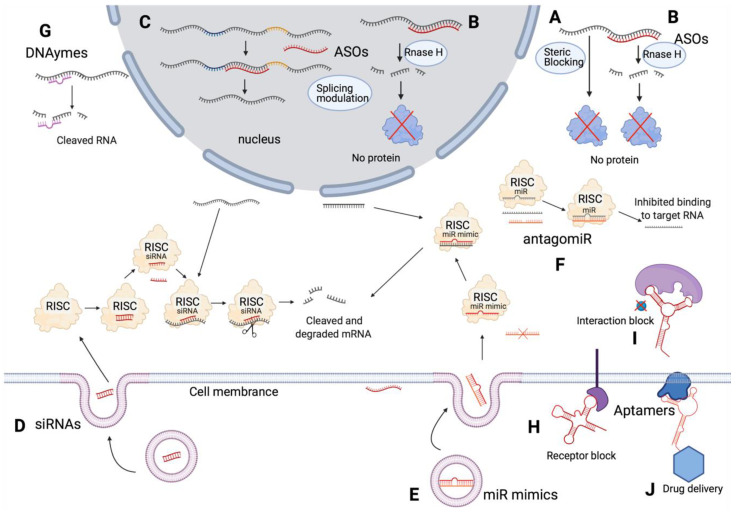
Summary of mechanisms of different types of oligonucleotide therapeutics. ASOs inhibit protein expression by sterically blocking translation (**A**) or RNase H dependent RNA degradation (**B**), or alter splicing (**C**); siRNAs degrade mRNA through RISC (**D**); miR mimetics degrade mRNA through RISC (**E**); antagomiRs block endogenous miRNA (**F**); DNAzymes specifically bind and cleave RNA (**G**); aptamers block cell receptors (**H**), block interactions between proteins (**I**), or act as drug delivery vectors (**J**). RISC, RNA-induced silencing complex. ASOs, aptamers and DNAzymes can also be delivered through vectors and cell endocytosis (not shown in the figure). Figure created with BioRender.com. Adapted from https://app.biorender.com/biorender-templates (accessed on 15 March 2021).

Currently, oligonucleotide therapeutics investigated in clinical trials include ASOs, siRNAs, miRNAs, aptamers, and DNAzymes. Their mechanisms of action differ, resulting in the correction of either abnormal expression or splicing patterns (Figure 2). Endocytosis is the main pathway for oligonucleotides to enter cells and various endocytic pathways are involved in the internalization of oligonucleotides [23]. Oligonucleotides can remain trapped in late endosomes, multivesicular bodies, and lysosomes, with a limited number being released from these endomembrane compartments to the cytoplasm and nucleus to enable their function [24]. Chemical modifications, conjugates and nanocarriers aid in overcoming such endosomal barriers [24]. For instance, different types of small molecules and anionic polymer conjugates are used to alter membrane stability of endosomes to release oligonucleotides [24]. 

### 2.1. Antisense Oligonucleotides (ASOs)

**Figure 3:**
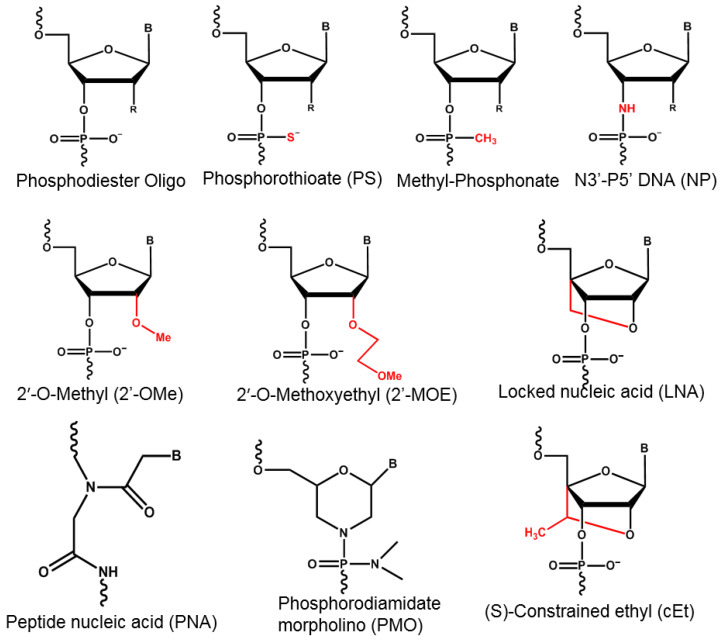
Overview of different chemical modification of antisense oligonucleotides.

ASOs are small single-stranded deoxyribonucleotides (~12–30 nt) that specifically bind target RNAs through Watson–Crick base pairing [25,26]. Unmodified ASOs contains phosphodiester backbones, which create challenges for cellular uptake and are easily degraded by endogenous nucleases [22,27,28]. Their blood circulating half-life is comparably short (less than 5 min) due to fast nuclease degradation and low affinity to plasma proteins [25,26,29,30]. Thus, the phosphodiester backbone is usually chemically modified to increase cellular uptake and endonuclease resistance (Figure 3) [31]. 

There are three generations of modified ASOs. First generation ASOs carry phosphorothioate (PS) backbones, where a non-bridging oxygen atom is replaced by a sulfur group, increasing resistance to nuclease degradation [25]. However, the affinity to target mRNAs is slightly reduced due to a decreased melting temperature of the PS ASO-mRNA complex [25,26]. This modification also increases the sequence length-dependent binding affinity to plasma proteins, which improves the blood circulating half-life in return, while toxicity is increased at high concentrations [32,33,34]. PS ASOs are taken up readily by mammalian cells with fast growing cells showing higher uptake efficiency compared to slow growing cells [35]. PS ASOs bind to cell surface proteins and enter cells through different forms of endocytosis including surface receptor mediated pathways and micropinocytosis [35]. Further, PS modifications also increase intracellular protein binding such as to nucleolin, which aids in the enrichment of PS ASO in the nucleus [27]. The non-bridging oxygen atom can also be replaced by a methyl or amine group, to generate methyl-phosphonate ASOs (MP ASOs) and N3′-P5′ phosphoramidates ASOs (N3′-P5′ NP ASOs), respectively (Figure 3) [36]. While MP ASOs show higher nuclease stability than PS ASOs, their reduced overall charge limit cellular uptake and their protein binding ability, leading to shorter blood circulating half-life compared to PS ASOs [30]. N3′-P5′ NP ASOs obtain increased affinity to target sequences and increased resistance to nuclease degradation, but exhibit low protein binding ability and are easy to degrade under acidic conditions, which may exist in solid tumors [25,26,37,38,39]. As one application of ASOs is to degrade target RNA by recruiting RNAse H, it should be noted that only the sulfur backbone modification of first generation ASOs retains RNAse H cleavage activity [40]. RNAse H is an endonuclease that can bind to RNA/DNA hybrids, RNA duplexes and DNA duplexes, but it only degrades RNA in RNA/DNA hybrids via a hydrolytic reaction [25,26,40,41]. Fomivirsen, the first FDA approved ASO, is an example of a PS ASO in the clinic [42]. 

Additional chemical modifications of ASOs further increase the binding affinity to target RNAs as well as nuclease resistance [43]. These developments include modifications at the 2′ position of the sugar moiety, replacing the hydroxyl group with methyl or methoxyethyl groups to form sugar-modified analogues such as 2′-O-methyl (2′-OMe) and 2′-O-methoxyethyl (2′-MOE) (Figure 3) [43]. These modifications also increase the toxicity [43]. ASOs with fully modified ribose backbones cannot undergo RNAse H dependent degradation [44]. Therefore, second generation PS ASOs intended for target degradation retain PS-modified deoxynucleotides in the central region to allow for RNase H cleavage, with 2′-MOE-PS and other sugar modifications at both 3′ and 5′ ends, resulting in a gapmer structure [45]. ASOs with fully modified ribose backbones can be used as steric blocking ASOs or splicing modulators [27]. Steric blocking ASOs bind to target RNA with high affinity, which can interfere with the interaction between target RNA and other molecules (RNA, DNA and proteins) important for its function [46]. Mipomersen and inotersen are second generation 2′-MOE-PS ASOs with 5′-methyl pyrimidines at both 3′ and 5′ ends [46]. 

Third generation ASOs utilize various modifications to further decrease nuclease degradation and increase target binding ability [47]. Locked nucleic acids (LNAs), in which the 2′-oxygen and the 4′-carbon on the same ribose are connected through a methylene bridge, show increased stability through increased melting temperatures (Figure 3) [48,49]. Similar to second generation ASOs, fully modified LNAs cannot activate RNase H cleavage [48,49]. To enable the RNA cleavage function, a gapmer structure with a central DNA region and LNAs at both ends is required [48]. LNAs have been reported to be associated with high liver toxicity, while introducing unmodified oligonucleotides in the gapmer structure was reported to reduce liver toxicity [50]. Constrained methyl- and ethyl-bridges in constrained methoxyethyl (cMOE) and constrained ethyl (cEt) oligonucleotides further reduce toxicity compared to LNA oligonucleotides [50]. Phosphorodiamidate morpholino oligomers (PMOs) and peptide nucleic acids (PNAs) are the other two most commonly used third-generation ASO [51]. In PMOs, a morpholine moiety connected through methylene phosphorodiamidate replaces the ribose (Figure 3). PMOs are frequently used to block mRNA translation [51]. In PNAs, which are frequently used in blocking transcription or translation, the deoxyribose phosphate backbone is replaced by a pseudopeptide backbone, which leads to an increase in melting temperature (Tm) and high affinity to DNA or RNA [52]. Since PMOs and PNAs are neutrally charged molecules, they do not show high levels of plasma protein binding and are rapidly cleared via renal pathways [27]. Currently, there are no third generation ASO that are approved by the FDA.

First generation ASOs (fully PS modified) are not widely used at this stage as ASOs with PS modifications usually utilize a gapmer structure with second or third generation modifications on the 5′ and 3′ ends. Such modifications increase target sequence binding affinity, increase resistance to nuclease, and reduce immune activation. However, some of these more recently developed modifications exhibit higher toxicity (Table 3). Further, ASOs are often conjugated with various moieties including cholesterol, peptides, sugar and aptamers for the purpose of either specific targeting or cell penetration [27]. An example is the N-acetylgalactosamine (GalNAc) conjugate, which binds to the asialoglycoprotein receptor (ASGPR) highly expressed in hepatocytes and leads to endocytosis of such modified ASOs [53]. Furthermore, polymer conjugates, such as cationic polymers or polyethylene glycol (PEG), improve resistance to nuclease degradation and increase cellular uptake [54].

### 2.2. CpG Oligonucleotides

CpG oligonucleotides are short single-stranded oligodeoxynucleotides containing CpG motifs and are used in the immunomodulatory response to stimulate Toll-like receptor 9 (TLR9) [55]. TLR9 plays a role in the recognition of pathogen molecules and the activation of the innate immune response [55,56]. The human Toll-like receptor family contains ten members, which recognize different types of molecules [55]. TLR9 subfamilies are mainly expressed in B cells and plasmacytoid dendritic cells (pDCs) and recognize unmethylated CpG-DNA [55]. CpG motifs interact with TLR9 and activate B cells and pDCs, which boosts the immune response, leading to proliferation and differentiation of these cells and cytokine secretion, and the activation of genes involved in inflammatory responses [57,58,59]. In response to chemotherapy, TLR9 acts as a tumor sensor by recognizing tumor released DNA, triggering an immune response and activating tumor-specific cytotoxic T lymphocytes (CTLs) [57]. This principle is exploited in the application of synthetic CpG oligonucleotides to enhance TLR9 related immune responses [57,58,59]. Currently, there are three types of CpG oligonucleotides being developed and tested in clinical trials, based on the types of immune cells activated: class A, B, and C. Class A CpG oligonucleotides (including ODN-2216) contain a single CpG motif and induce IFN-α secretion from pDCs [60]. Class B CpG oligonucleotides (including CpG-7909, IMO-2055, and 1018-ISS) contain multiple CpG motifs and stimulate B-cell activation and pDC maturation [61]. Class C CpG oligonucleotides (including SD-101) combine the characteristics of both class A and B and can induce high levels of IL-12 secretion from pDCs [62]. CpG oligonucleotides are being tested both as monotherapy and immunotherapeutic adjuvants in combination with chemotherapy, radiotherapy, and targeted therapy [63].

### 2.3. miRNAs

miRNAs are short (~18–25 nt) single stranded non-coding RNAs that contain complementary sequences to one or more mRNAs, usually within the 3′-UTR [64]. They play an important function in post-transcriptional gene repression [64]. miRNA genes are transcribed in the nucleus by RNA polymerase II to form pri-miRNAs, which are cleaved by RNase III to form pre-miRNAs and are exported into the cytoplasm [65,66]. The pre-miRNAs are further processed by Dicer to form double stranded miRNAs, which are components of the RNA-induced silencing complex (RISC) [65]. 

Dysregulated expression of many miRNAs is found in various types of cancers due to altered miRNA gene copy numbers, changes to the epigenetic status of miRNA genes, or aberrant transcription or synthesis [67]. Different types of cancers were shown to have different miRNA signatures compared to normal tissues [68]. Moreover, specific miRNA signatures are associated with drug resistance, such as elevated miR-214 levels in recurrent cisplatin-resistant patients [69]. Therefore, miRNAs and in particular circulating miRNAs may have potential as cancer biomarkers or indicators of drug sensitivity. Most current clinical trials on miRNAs (101 of 108) aim to discover novel cancer biomarkers or indicators of drug sensitivity (Supplementary Materials, miRNA screening). Around 30% of the trials investigate miRNAs that have previously been identified to show altered expression in cancer and the remaining 70% intend to find novel miRNA signatures for diagnostic use (Supplementary Materials, miRNA screening). Early studies using naked forms of miRNAs were often unsuccessful due to challenges with degradation and cell delivery [70]. Structure modifications and delivery systems including nanoparticles may aid in overcoming these obstacles [70].

### 2.4. siRNAs

SiRNAs are 20–25 bp long double stranded RNAs, which are produced by Dicer from long dsRNAs and small hairpin RNAs [71]. Similar to miRNAs, siRNAs work in complex with RISC to post-transcriptionally silence target gene expressions [71]. siRNAs have been extensively used in RNA interference and represent another strategy to modulate overexpressed mRNAs in cancer [72]. siRNAs are cleaved by endonucleases at pyrimidines and by exonucleases at both ends [73]. Chemical alterations of siRNAs such as non-consecutive 2′-OMe modifications and PS modifications enhance nuclease resistance and selectivity of RISC loading without inhibiting RNAi efficiency [74]. siRNAs are usually delivered using lipid nanoparticles, which protect the encapsulated siRNAs and improves cellular uptake by macropinocytosis [35]. Moreover, various types of nanoparticles and conjugates have been developed for safe and targeted delivery of siRNAs [75]. These either target specific cell surface receptors, increase non-specific interactions with cell membranes, or assist with endocytosis [73]. Currently, most siRNAs in clinical trials are focused on genetic diseases.

### 2.5. Aptamers

Aptamer oligonucleotides are single-stranded DNA or RNA oligonucleotides that bind targets with high affinity based on their three-dimensional structures [17]. Aptamer oligonucleotides are screened using the systematic evolution of ligands by exponential enrichment (SELEX) technology from a synthetic oligonucleotide pool against a target (either RNA or protein) [76]. Aptamers are often used as a drug delivery system for chemotherapeutics, RNA therapeutics and nanoparticles containing drugs by specifically binding to cell- and tumor-type specific cell surface receptors [44]. Aptamers can also act as therapeutics by blocking essential interactions between the target and other molecules [44]. In addition, aptamers can be used as antagonists of immune checkpoints such as PD-1 and PD-L1 to remodel immune responses, or as agonists of costimulatory receptors to activate related pathways [77]. Moreover, aptamers have been used in cancer diagnosis and detection due to their high affinity to targets resulting from the SELEX selection process [78]. Aptamers are selected to target cancer surface markers such as nucleolin and MUC1 or metastatic cell lines to detect various types of cancer or cancer metastasis [78]. Unmodified DNA/RNA aptamers are nuclease sensitive with poor serum stability in vivo [79]. Modifications on the sugar ring and phosphodiester linkage have been shown to increase nuclease resistance. Moreover, modifications on the 5′ and 3′ end (end capping) and the utilization of an L-enantiomer form of nucleic acids provide additional options to protect aptamers from nuclease degradation [80]. To increase the half-life in systemic circulation, attachments on the 5′ end including cholesterol, dialkyl lipids and PEG reduce renal filtration and prolong circulation time [80]. While showing promising results in vitro, most aptamers currently in preclinical development as cancer therapeutics target cell surface receptors including CD28, CD40, and 4-1BB (CD137), and have not entered clinical trials yet [81].

### 2.6. DNAzymes 

DNAzymes are catalytically active DNA oligonucleotides that have the potential to cleave RNA and to ligate and phosphorylate DNA or RNA [82,83]. Currently, DNAzymes are not found in nature but are generated using in vitro selection strategies (SELEX) [83]. DNAzymes that cleave RNAs are the most well studied type of DNAzymes in cancer research due to their potential to silence genes [84]. These RNA-cleavage DNAzymes consist of a catalytic core and two arms, which bind and recognize specific RNA sequence through Watson–Crick base pairing [85]. Two widely studied DNAzymes are 10-23 DNAzymes and 8-17 DNAzymes [86]. The efficiency of the RNA cleavage relies on the metal ion in the core [84]. DNAzymes also face the challenge of nuclease degradation. Chemical modifications such as phosphorothioate (PS) and 2′-O-methoxyethyl (2′-MOe) and conjugates including polyamines and peptides significantly increase the stability of DNAzymes against nucleases in vivo [85,87]. Efficient cellular delivery is another barrier for the application of DNAzymes and nanocarriers such as lipid nanoparticles and cationic polymers can improve uptake [88]. Despite promising outcomes in vitro and in animal models, only one clinical trial has been completed for DNAzymes in oncology thus far [84]. 

## 3. Clinical Trials for Oligonucleotide Therapeutics in Oncology

For the purpose of this review, we summarized clinical trials registered on ClinicalTrials.gov by searching terms including oligonucleotide, antisense oligonucleotide (ASO), small interfering RNA (siRNA), microRNA (miRNA), aptamer, and DNAzyme in the intervention/treatment section with diseases limited to cancer/neoplasm/tumor/carcinoma. We also investigated pipelines and products of some of the major nucleic acid therapeutics companies including Arrowhead Pharmaceuticals, Akcea Therapeutics, Alnylam Pharmaceuticals, BioNTech, CureVac, Gotham Therapeutics, Gradalis, Ionis Pharmaceuticals, Moderna, and Quark Pharmaceuticals. We retrieved a total of 229 clinical trials for oligonucleotide therapeutics in oncology. 

### 3.1. Clinical Trials for ASOs in Oncology

There are 120 trials completed thus far, which consist of 44 phase 1 trials, 23 phase 1/2 trials, 44 phase 2 trials, and nine phase 3 trials. According to ClinicalTrials.gov, there are currently 35 terminated trials, five withdrawn, one suspended, six with unknown status and 27 ongoing trials (Supplementary Materials, ASOs). Here, we summarize landmark studies and the most recent trials for ASOs in oncology since 1993. 

#### 3.1.1. First ASO in Cancer Clinical Trials, G4460

The first clinical trial of ASOs in oncology registered on ClinicalTrials.gov is a phase 2 trial launched in 1993, investigating G4460 (a PS ASO developed by Genta Incorporated) in chronic myelogenous leukemia (clinical trial identifier: NCT00002592). G4460 binds to codon sequences 2–9 of the *CMYB* mRNA and triggers RNAse H dependent degradation [89]. *CMYB* is a proto-oncogene that encodes a transcription regulator essential for cell proliferation, differentiation, and apoptosis [90,91]. Over-expression of c-Myb leads to increased proliferation and reduced differentiation in many types of cancers including leukemias and breast cancers [91]. A preclinical experiment indicated that the ASO inhibits proliferation of the human leukemia cell line HL-60 by 75%–80% [92,93]. In this trial, tumor cells were removed from bone marrow transplants by treating the bone marrow with G4460. Results were not disclosed. However, a phase 1 trial by the same investigator, Selina M. Luger, investigates G4460 in other hematologic malignancies, using a similar method as NCT00002592 (clinical trial identifier: NCT00780052). A publication for NCT00780052 reports that *CMYB* mRNA levels were undetectable in eight out of 18 (44.4%) of the treated bone marrow samples and major cytogenetic responses were found in six of 14 (42.9%) patients with ASO treated bone marrow transplants [94]. Clinical efficacy of the ASO could not be evaluated in this phase 1 trial, but the study demonstrated the feasibility of transplanting ASO treated bone marrow [94]. 

#### 3.1.2. ASOs in Phase 3 Trials

There are 15 phase 2/3 or phase 3 trials, 10 were completed, three were terminated, one was withdrawn and two are of unknown status (Supplementary Materials, ASOs). These trials are for the ASO drugs Genasense (targeting BCL2, eight trials), ISIS-3521 (targeting PKCα, two trials), AP-120009 (targeting TGFB2, one trial) and OGX-011 (targeting ApoJ, four trials). 

##### B-Cell Lymphoma 2 (BCL2) 

Since 1997, 44 clinical trials have been performed on Genasense (developed by Genta Incorporated) in various types of cancers such as melanoma, leukemia, multiple myeloma, plasma cell cancer, and lung cancer. Moreover, 17 trials were launched by Genta (Supplementary Materials). Genasense, also named oblimersen and G3139, is a PS ASO targeting the first six codons of *BCL2* mRNA and triggering RNase H dependent degradation [95]. B-cell lymphoma 2 (BCL2) is a 26 kDa protein that maintains the integrity of the outer mitochondrial membrane [96]. BCL2 regulates the mitochondrial apoptosis pathway and overexpression leads to escape from apoptosis in cancer cells [97,98]. Genasense obtained orphan drug designation for chronic lymphocytic leukemia (CLL) in 2001 [95]. Of eight initiated phase 3 trials, five were completed. However, Genasense was not approved because of unsatisfactory primary endpoint outcomes in all phase 3 trials, as overall survival (OS) and progression-free survival (PFS) did not increase with statistical significance. The secondary endpoint was reached for NCT00024440, which investigated fludarabine plus cyclophosphamide alone vs. combined with Genasense in CLL, showing complete or partial response (*p* = 0.025) in the Genasense combination group [99]. Three trials investigating different combinations of chemotherapy with Genasense were terminated since the production of Genasense ceased (NCT00064259, NCT00736450, and NCT01200342). 

There are several phase 1 or 2 trials investigating different ASOs targeting *BCL2*, including BP1002 developed by Bio-Path (NCT04072458), PNT2258 developed by ProNAi Therapeutics (NCT01191775, NCT01733238, NCT02226965), and SPC2996 developed by Santaris Pharma (NCT00285103). BP1002 is a liposome encapsulated neutral P-ethoxy-ASO that blocks the translation of *BCL2* mRNA. PNT2258 is also a liposome encapsulated ASO but targets the *BCL2* protomer and inhibits transcription by DNA interference. A phase 1 trial confirmed its safety [100]. A phase 2 trial of PNT2258 (NCT01733238) indicated a single-agent response rate of 8.1% in diffuse large B-cell lymphoma (DLBCL) patients, prompting ProNAi Therapeutics to suspended further development due to low efficacy, even though it was granted orphan drug designation for treating DLBCL [101]. SPC2996 is a gapmer LNA targeting codons 1–6 of the *BCL2* mRNA and differs in just three nucleotides from the Genasense sequence [102]. The phase 1/2 trial of SPC2996 (NCT00285103) indicated that 40% of the patients underwent painful inflammatory reactions and failed to prove significant single-agent efficacy to inhibit BCL2 expression, similar to Genasense [102].

The failure of ASOs to target *BCL2* may be due to its long protein half-life, which reduces the continuing inhibition of BCL2 activity. The focus of BCL2 research has since shifted to inhibit domains of the BCL2 protein [103]. In 2016, a small molecule inhibitor targeting the BH3 domain of BCL2, venetoclax, was approved by the FDA due to a significant response rate (>70%) in a phase 2 trial [104]. 

##### Protein Kinase C Alpha (PKCα)

Protein kinase C alpha (PKCα) is a serine/threonine kinase that is involved in diverse cellular signaling pathways [25,26,105,106]. Overexpression of PKCα and the PKC family overall increases cancer cell proliferation and has been shown to correlate with progression of several types of cancer, such as breast and ovarian cancers [106,107]. ISIS-3521, also known as LY900003, aprinocarsen and Affinitak, is a PS ASO initially developed by Ionis Pharmaceuticals and licensed to Eli Lilly for co-development, that targets the 3′ UTR of *PKCA* mRNA, resulting in the inhibition of PKCα protein synthesis [108]. Phase 1 and 2 trials of ISIS-3521 in various cancer types demonstrated mild to moderate toxicity and low single-agent activity [108]. Combinations of chemotherapy and ISIS-3521 in phase 2 trials in lung cancer showed acceptable response rates, resulting in the initiation of two phase 3 combination trials [108,109]. ISIS-3521 received Fast Track Designation, but the phase 3 trials on non-small cell lung cancer showed that ISIS-3521 combined with chemotherapy did not increase OS and failed other clinical measures, including low response rates and increased toxicity (*p* < 0.0001). The ASO drug was thus not approved, and Eli Lilly announced no further development [110,111]. 

##### Transforming Growth Factor Beta (TGFβ)

Transforming growth factor beta (TGFβ) is a secreted cytokine that has three isoforms, β1, β2, and β3, and plays important roles in embryonic development, cell growth and differentiation [112]. Overexpression of TGFβ leads to increased cell proliferation, invasion and metastasis, and has been observed in various cancers including glioma, pancreatic and colorectal cancers [113,114]. AP-120009, also named trabedersen and OT-101, is a PS ASO developed by Antisense Pharma (now Isarna Therapeutics), that targets *TGFB2* mRNA and induces RNAse H dependent degradation [115]. Preclinical trials indicated that AP-120009 significantly reduces TGFβ2 expression in cancer and exhibited low toxicity in animal models [25,26,116,117]. Phase 1 and 2 trials demonstrated a favorable safety profile for AP-120009 and extended patient survival, especially for anaplastic astrocytoma patients with two complete responses [116]. A phase 2b trial for AP-120009 further indicated significant cancer control rates (drug response) in the anaplastic astrocytoma (AA) subgroup with AP-120009 compared to AA patients receiving chemotherapy (*p* = 0.0032) [118]. The FDA granted orphan drug designation for AP-120009 in 2002 for diffuse intrinsic pontine glioma (DIPG) and in 2009 for pancreatic cancer [119]. The phase 3 trial of AP-120009 on anaplastic astrocytoma and glioblastoma patients was terminated by Isarna Therapeutics. Further development of AP-120009 is still ongoing and Mateon Therapeutics/Oncotelic announced in 2020 that a combination of AP-120009 and interleukin-2 (IL-2) will be tested in phase 1 trials.

##### Apolipoprotein J (ApoJ)

Clusterin, or apolipoprotein J (ApoJ), is a multifunctional glycoprotein acting as a cell-aggregating factor and playing an important role in cell adhesion and programmed cell death [120]. Studies showed that it is overexpressed in metastatic cancer cells including colon, bladder, and hepatocellular cancer [121]. The overexpression of ApoJ increases cell migration and assists cancer metastasis [121]. In addition, overexpression of ApoJ reduces TNFα-mediated apoptosis and is associated with the development of drug resistance [122]. OGX-011, a 2′-MOE PS ASO co-developed by Ionis Pharmaceuticals and OncoGenex Pharmaceuticals, targets the translation-initiation site of ApoJ [123]. OGX-011 received Fast Track Designation, but two of its phase 3 trials failed [123]. The phase 3 trials NCT01578655 and NCT01188187 investigated the combination of OGX-011 with abazitaxel/docetaxel and prednisone versus chemotherapy alone in metastatic castration-resistant prostate cancer. No difference of OS was found when comparing chemotherapy + OGX-011 with chemotherapy alone in both NCT01578655 and NCT01188187 [123,124,125]. Serious adverse events were detected in both patient cohorts, in 49% and 42% of patients in each group in NCT01578655, and in 43% and 36% in NCT01188187 [123]. 

#### 3.1.3. Additional ASOs in Phase 2 Trials

There are additional ASOs that have reached phase 2 trials, with AEG35156 (*XIAP*), danvatirsen (*STAT3*), apatorsen (*HSP27*), ISIS 2503 (*HRAS*), ISIS 183750 (*EIF4E*) and cenersen (*P53*) making up the largest number of trials [25]. While all preclinical studies provided significant anti-tumor responses, the clinical trials have various levels of responses in patients. Three ASOs that have been tested in several phase 2 trials are discussed below. 

##### X-Linked Inhibitor of Apoptosis (XIAP)

*XIAP* encodes the X-linked inhibitor of apoptosis protein, which directly binds caspases to inhibit apoptosis [126]. XIAP is overexpressed in a variety of cancers and drives resistance to chemotherapeutics and targeted drugs [126]. AEG35156 is a PS ASO developed by Aegera Pharmaceuticals that triggers RNase H dependent degradation of *XIAP* mRNA. In a phase 2 trial combined with sorafenib in advanced hepatocellular cancer, AEG35156 showed higher objective response rates (ORRs) in combination groups compared to the sorafenib only group and was accompanied by few serious adverse events (1 in 32) [127].

##### Signal Transducer and Activator of Transcription 3 (STAT3)

Signal transducer and activator of transcription 3 (STAT3) is a transcription factor that participates in cellular proliferation, migration, invasion and survival [128]. Activation of STAT3 has been found in various cancer types and inhibition of STAT3 leads to decreased proliferation in cancer [129]. Danvatirsen (AZD9150, ISIS STAT3Rx) is a 16 nt cEt modified PS ASO with cEt nucleosides at both 5′ and 3′ ends developed by Ionis Pharmaceuticals that sterically blocks translation of *STAT3* mRNA [130]. Danvatirsen demonstrated promising anti-tumor activity in cell and animal models [129,131]. A phase 1b trial (NCT01563302) demonstrated two complete responses and two partial responses in DLBCL patients (total 30) [132]. Phase 2 trials are currently recruiting.

##### Heat Shock Protein 27 (HSP27)

Heat shock protein 27 (HSP27) is a 27-kDa protein induced by stress such as heat, hyperthermia and chemotherapy. HSP27 acts as a protein chaperone, is involved in the inhibition of apoptosis and is ubiquitously expressed [133]. HSP27 is overexpressed in many cancer types and associated with poor prognosis, cancer cell survival and drug resistance [134]. Apatorsen (OGX-427) is a 2′-MOE PS ASO developed by OncoGenex Pharmaceuticals and triggers *HSP27* mRNA degradation via RNase H. Apatorsen inhibits the expression of HSP27 to increase the sensitivity of cancer cells to chemotherapy [135]. Preclinical experiments indicate that apatorsen combined with erlotinib inhibits growth of A549 xenografts significantly, leading to high apoptosis rates compared to controls [135]. However, three phase 2 trials (NCT01844817 for pancreatic cancer, NCT01829113 for non-squamous-non-small-cell lung cancer, NCT01120470 for prostate cancer) investigating apatorsen combined with chemotherapy did not significantly improve either PFS and OS (NCT01120470 and NCT01844817), or disease response (NCT01829113) in cancer patients without HSP27 level screening (*p* > 0.05) [136,137,138].

In summary, significant responses of ASOs as therapeutics in oncology were achieved in subgroups of patients in phase 3 trials for Genasense and trabedersen, and some responses were seen in AEG35156 and danvatirsen. Thus far, a number of phase 3 clinical trials of ASOs in cancer have not reached sufficient primary endpoints such as PFS and OS, suggesting that future trials may want to focus on determining the factors underlying the heterogeneous response to the drugs, specifically evaluating pre-screened subgroups of patients, and focusing on new targets. In addition, most ASOs that have completed phase 3 trials thus far are first generation ASOs (PS ASOs), with drawbacks including reduced affinity and specificity, which may contribute to somewhat low clinical efficacy in clinical trials [139]. Future clinical trials with second or third generation ASOs may lead to increased efficacy and improved PFS and/or OS.

### 3.2. CpG Oligonucleotides 

Thus far, just one CpG oligonucleotide has completed phase 3 trials, CpG 7909. CpG 7909 (PF03512676) is a class B 24 nt agonist of TLR9 in B cells and pDCs [140]. Both phase 3 trials of CpG 7909 (PF03512676) in non-small-cell lung carcinoma showed no improvement of OS and increased adverse events over control groups [141,142,143]. Two additional CpG oligonucleotide therapeutics have completed phase 2 trials, IMO-2055 (EMD 1201081) and 1018 ISS, and a third, SD-101, completed phase 1 trials. The phase 2 trial on IMO-2055 (EMD 1201081) in combination with cetuximab (NCT01040832) showed acceptable toxicity in squamous cell cancer of the head and neck patients but low clinical efficacy (no significant improvement of PFS) compared to the control group on cetuximab alone [144]. 1018 ISS is a 22 bp unmethylated class B CpG PS oligodeoxynucleotide. A phase 2 trial (NCT00251394) in combination with rituximab for non-Hodgkin lymphoma showed an overall response rate of 32% with low toxicity [145]. For SD-101, the phase 1b trial combined with pembrolizumab in advanced melanoma showed that the combination is well tolerated and induced an ORR of 78% in patients without prior PD-1 treatment experience, but an ORR of 15% in patients with prior PD-1 treatment experience [146]. CpG oligonucleotides usually serve as adjuvants to improve the effects of chemotherapeutics or targeted therapy, and further evidence is needed for the use of CpG oligonucleotides as an immunologic adjuvant [63].

### 3.3. miRNAs

Two types of oligonucleotides are currently being used as miRNA therapeutics: miRNA mimetics to restore the levels of miRNAs downregulated in cancer, and antagomiRs to inhibit overexpressed miRNAs [147] (Figure 2). Thus far, six clinical trials have been investigating their potential as oncology drugs. Of these, three were terminated and one withdrawn, leaving two phase 1 trials completed and one still recruiting patients. The termination of NCT01829971 and the withdrawal of NCT02862145 were due to frequent related serious adverse events reported by the investigators, and the other two terminated trials were due to business reasons and patients placed in other trials according to comments on ClinicalTrials.gov (Supplementary Materials, miRNA).

One phase 1 trial (NCT02580552) investigated cobomarsen (MRG-106), an LNA blocking *MIR155* (associated with poor prognosis), in lymphoma and leukemia. In this phase 1 trial, patients showed acceptable toxicity and drug responses in all four patients recruited for intratumoral injection with decreased neoplastic cell density and depth [148]. The FDA granted Orphan Drug Designation to cobomarsen for mycosis fungoides type cutaneous T-cell lymphoma in 2017. *In vitro* studies indicate that depleted miR-16 levels can be restored with *MIR16* mimetics in malignant pleural mesothelioma cell lines, which led to growth inhibition [149]. However, the phase 1 trial of TargomiRs (*MIR16* based miRNA mimetic) showed low response rates (one in 22 patients) [149,150].

All miRNA therapeutics across indications are still in clinical trials with some positive outcomes thus far. Studies on miRNAs for cancer diagnosis have exponentially increased over the past 20 years and miRNA based diagnostic tools are being developed and tested by companies such as Rosetta Genomics, Interpace Diagnostics and Hummingbird Diagnostics [147].

### 3.4. siRNAs

Currently, there is one phase 2 trial recruiting patients using siRNAs as therapeutics in oncology. NCT01676259 is investigating siG12D-LODER, a novel biodegradable polymeric matrix containing an siRNA targeting mutated *KRAS* (KRAS G12X mutation) in advanced pancreatic cancer patients undergoing chemotherapy [151]. In this trial, siG12D-LODER is delivered directly into the solid tumor through implantation. The trial was scheduled to complete in June 2020, but the results have not yet been released. A phase 1 trial (NCT01188785) showed a decrease in tumor marker CA19-9 in 70% of the patients implanted with siG12D-LODER, but no difference in OS between all trial groups (different dosage of siG12D-LODER) [152]. As to other therapeutics developed targeting mutant *KRAS*, the FDA granted sotorasib, a KRAS G12C small molecule inhibitor priority review status in 2021 for the treatment of non–small cell lung cancer patients with KRAS G12C mutation. Most trials assessing siRNA drugs for cancer are currently in phase 1. However, some trials show promising initial responses. In NCT01158079, which tests a lipid nanoparticle containing two siRNAs targeting two genes (VEGF and kinesin spindle protein KSP) administered through intravenous infusion, a complete response was seen in one patient and disease stabilization for 1–1.5 years in several other patients [152,153]. In NCT02110563, which used siRNAs targeting the oncogene *MYC*, complete response was seen in one patient and tumor shrinkage was observed in several patients [154]. Similarly, NCT00672542, which utilized intradermal injection of siRNA transfected dendritic cells for melanoma patients resulted in one complete and one partial response (n = 5) [155].

### 3.5. Aptamers

There are nine trials for four aptamer drugs on ClinicalTrials.gov (six completed, one terminated and two with unknown status) (Supplementary Materials, Aptamers). Four trials investigate the drug AS1411, a DNA aptamer targeting nucleolin, with one phase 2 trial (NCT00740441) indicating limited response in one patient (*FGFR2* and *mTOR* mutations) based on high levels of reduction in tumor diameter [156]. Three trials evaluated NOX-A12, an RNA aptamer that neutralizes *CXCL12*, a chemokine regulating the activity of CLL cells, with phase 2 trial NCT01486797 showing promising response rates (86%) [157,158]. A trial for the drug 68Ga-Sgc8, a DNA aptamer targeting protein tyrosine kinase-7 (PTK7) is used for diagnostic purposes in colorectal cancer (status: unknown). Lastly, a trial for the drug EYE001 (anti-VEGF pegylated aptamer) has not released any results yet.

### 3.6. DNAzymes 

Thus far, just one trial for a DNAzyme in oncology is logged on ClinicalTrials.gov. NCT01449942 (Phase 1 and 2) utilized a DNAzyme to reduce the expression of Epstein-Barr virus—encoded latent membrane protein 1 (EBV-LMP1), which is an oncogenic protein in nasopharyngeal carcinoma [159]. A significant decline of K_trans_ values, which indicates tumor blood flow and permeability, was found in the experimental group compared to the control group. The secondary endpoint parameters k_ep_ (the rate of contrast agent transfer to plasma) and v_e_ (distribution volume of contrast agent) showed no significant difference between groups [159]. A phase 1 trial in 2010 (not registered on ClinicalTrials.gov) of the DNAzyme Dz13, which targets c-Jun, showed no serious adverse effects in patients with nodular basal-cell cancer, and c-Jun expression was decreased. However, there were no further clinical trials to confirm the efficacy of the treatment [160]. c-Jun is an important target of the Jun N-terminal kinase (JNK) signaling pathway, and several JNK inhibitors have been approved by the FDA [161]. Meanwhile, at least seven clinical trials were performed on the DNAzyme hgd40 (SB010, SB011, and SB012), which targets GATA3, a transcription factor highly mutated in more than 10% of breast cancers, in patients with severe allergic bronchial asthma, atopic dermatitis, and ulcerative colitis [162,163]. 

Thus far, ASOs are the most well-studied oligonucleotide therapeutics modality in clinical trials for oncology purposes, with the largest number of registered trials. Other nucleic acid drugs modalities such as miRNAs, siRNAs, aptamers, and DNAzymes are all at early stages of clinical trials in cancer, with promising responses in some patients.

## 4. Benefits of Oligonucleotide Therapeutics 

Oligonucleotide therapeutics are able to target previously ‘undruggable’ targets such as *RAS* and *MYC*, which contain large protein-protein interaction surfaces and/or lack suitably deep protein pockets, providing new approaches for complex diseases [46,164]. The approval of 13 oligonucleotide therapeutics by the FDA demonstrates their potential in a range of indications. For oncology, there are three ASOs that have been granted orphan drug designation.

With research exploring the underlying molecular mechanisms of diseases, oligonucleotide therapeutics represent a straight-forward drug design approach compared to small molecules and other targeted therapeutics. In addition, oligonucleotides can be synthesized in a shorter time frame and at lower cost compared to chemotherapeutics and other types of targeted therapies [165]. Oligonucleotide therapeutics show high affinity to targets based on one dimensional sequence matches, suggesting simplicity in design compared to time consuming large scale computational searches, and do not require validation of tertiary protein domain matches like small molecule therapeutics [46,165,166,167]. The comparably simple structure and reproducible chemistry of oligonucleotide backbones result in known safety profiles, such as reduced activation of an immune response, which is ideal for the usage in combination therapy targeting different genes [168]. 

ASOs are the most frequently studied oligonucleotide therapeutics in clinical trials currently. Various types of administration can be used for ASOs to increase the number of relevant clinical applications [25]. Variation in biodistribution and bioactivity of ASOs is largely driven by their backbone chemistry, which can be improved or changed by structure modifications and linkage to delivery vectors [25]. In contrast, siRNA and miRNA therapeutics to trigger endogenous RISC-dependent RNA silencing require suitable vectors for delivery, such as liposomes [169]. Aptamers have potential to be used extensively in multiple ways in oncology, including as therapeutics, diagnostics, and drug delivery vectors. 

## 5. Overcoming Challenges of Oligonucleotide Therapeutics 

Though most clinical trials of oligonucleotide therapeutics did not show significantly improved PFS, complete or partial response have been observed in many patients mentioned as discussed above [166,167,170,171]. Below, we discuss several factors that may impact the therapeutic efficacy of oligonucleotide therapeutics in clinical trials and suggest pathways to overcome current challenges. 

### 5.1. Drug Delivery Efficiency

Early clinical trials using unmodified or minimally modified oligonucleotide therapeutics (PS ASOs) faced challenges including efficiently delivering the drugs to cancer cells in the body [46]. Drugs need to overcome several barriers to successfully enter cells and avoid rapid clearance from circulation to exhibit therapeutic effects [24]. The review by Khvorova and Watts (2017) pointed out that applying these first-generation oligonucleotide therapeutics in trials may have been detrimental to the field [47]. Recent developments in nucleic acid chemistry and new modes of delivery seem to be able to overcome the delivery challenges earlier generation oligonucleotide therapeutics have faced, based on promising initial results indicating greatly improved clinical efficacy [47]. In addition to the second and third generation ASO backbone modifications discussed above, most types of oligonucleotides can be conjugated to small size molecules including lipids, peptides, or antibodies to enhance cellular uptake and promote cell specific targeting [46]. Ionis Pharmaceuticals’ ligand-conjugated technology is one example, linking 2′-MOE ASOs to triantennary N-acetylgalactosamine for specific targeting of hepatocytes through asialoglycoprotein receptors [172]. Drug delivery can also be improved with nanocarriers such as lipid and polymeric nanoparticles, spherical nucleic acids, DNA nanostructures, lipoplexes, liposomes and exosomes [46,166,167,173,174]. For instance, nanoparticles protect encapsulated siRNAs from endogenous nuclease cleavage and assist in cellular uptake. Aptamer conjugates are another efficient approach to precisely direct oligonucleotide therapeutics [78].

### 5.2. Complexity of Cancer 

All currently approved oligonucleotide therapeutics treat indications caused by mutations in a single gene, either causing dysregulated expression or aberrant splicing patterns. Thus, targeting the affected gene restores expression or corrects splicing patterns, leading to efficient treatments or even cures in some cases. However, cancer is a multifactor disease usually involving multiple genes, and targeting one of them may not be sufficient [175]. This is a problem not only for oligonucleotide therapeutics, but for all targeted treatments. Targeting multiple affected genes simultaneously may provide an interesting new therapeutic avenue [173,174]. Preclinical studies for targeted therapy have already provided evidence that targeting several pathways simultaneously may lead to a promising anti-cancer effect [166,167]. Most oligonucleotide therapeutics in clinical trials target one gene combined with chemotherapy. We note that one of the siRNA phase 1 clinical trials discussed above showed promising outcomes with several high response cases [153]. The drug tested contains two siRNAs targeting different genes (VEGF and KSP), which inhibit blood supply and proliferation of cancer cells at the same time [153]. Combination therapy targeting multiple affected genes may be a feasible approach in the future. Oligonucleotide therapeutics may be especially well suited for combination therapy as the same drug modality can be applied to target multiple cancer drivers at once. 

### 5.3. Drug Interactions

A number of clinical trials investigated the combination of oligonucleotide therapeutics with chemotherapy and reported no significant improvement of drug response and OS. As mentioned in Section 2.1, plasma proteins such as albumin play an important role as carrier for drug delivery and cellular uptake [176]. ASOs are usually modified to increase their half-life and affinity to plasma proteins and nearly all blood circulating PS ASO bind to plasma proteins [177]. In contrast, PNAs and PMOs exhibit lower plasma protein affinity (less than 25%) [178]. Other oligonucleotides such as siRNAs also require albumin transportation [176]. However, the plasma binding affinity of chemotherapeutics is significantly higher, ranging from >90% for carboplatin and paclitaxel to <30% for cyclophosphamide and cytarabine [179]. Thus, it is possible that oligonucleotides compete with chemotherapeutics for plasma protein binding, which may lead to reduced in vivo efficacy of the combination compared to chemotherapeutics alone [180]. Carrier based combination drug delivery systems using water-soluble polymer conjugates, nanoparticles, dendrimers, and liposomes provide advantages over simultaneous delivery in mixtures or sequential delivery of multiple drugs [181]. The use of polymeric nanoparticles for combination delivery of chemotherapeutics and siRNAs has shown some success [39].

## 6. Conclusions

Oligonucleotide therapeutics for oncology utilize their specific high affinity binding to target abnormally expressed or spliced genes that drive tumorigenesis and cancer progression. Oligonucleotide therapeutics exhibit promising outcomes in preclinical experiments and in some clinical trials. However, limited data are currently available on phase 3 trials. Recent advances in the development of oligonucleotide therapeutics show both improved efficacy and safety profiles, and enable drug delivery to specific cell types. In summary, oligonucleotide therapeutics provide a promising new approach for the treatment of cancer.

## Figures and Tables

**Table 1:** Summary of currently approved oligonucleotide therapeutics.

Drug Names	Market Names	Companies	FDA Approved	Indications	Drug Modality	Mechanisms	Targets
fomivirsen	Vitravene	Ionis Pharmaceuticals, Novartis	1998	Cytomegalovirus (CMV) retinitis	ASO	Translation block	CMV protein IE2
pegaptanib	Macugen	OSI Pharmaceuticals	2004	Neovascular age-related macular degeneration	Aptamer	Binding and blocking	Heparin-binding domain of VEGF-165
mipomersen	Kynamro	Kastle Therapeutics, Ionis Pharmaceuticals, Genzyme	2013	Homozygous familial hypercholesterolemia	ASO	RNase H degradation	Apolipoprotein B100
eteplirsen	Exondys 51	Sarepta Therapeutics	2016	Duchenne muscular dystrophy	ASO	Splicing modulation	Exon 51 of *DMD*
nusinersen	Spinraza	Ionis Pharmaceuticals, Biogen	2016	Spinal muscular atrophy	ASO	Splicing modulation	Exon 7 of *SMN2*
defibrotide	Defitelio	Jazz Pharmaceuticals	2016	Veno-occlusive disease in liver	Aptamer	Binding and activating	Adenosine A1/A2 receptor
inotersen	Tegsedi	Akcea Therapeutics	2018	Polyneuropathy caused by hereditary transthyretin-mediated (hATTR) amyloidosis	ASO	RNase H degradation	Transthyretin
milasen	Not applicable	Boston Children’s Hospital	2018	Mila Makovec’s CLN7 gene associated with Batten disease	ASO	Splicing modulation	*CLN7*
patisiran	Onpattro	Alnylam	2018	Polyneuropathy caused by hATTR amyloidosis	siRNA	RNAi	Transthyretin
golodirsen	Vyondys 53	Sarepta Therapeutics	2019	Duchenne muscular dystrophy	ASO	Splicing modulation	Exon 53 of *DMD*
givosiran	Givlaari	Alnylam	2019	Acute hepatic porphyria (AHP)	siRNA	RNAi	5-aminolevulinic acid synthase
volanesorsen	Waylivra	Akcea Therapeutics	EMA approved in 2019 ^1^	Familial chylomicronemia syndrome (FCS)	ASO	RNase H degradation	Apolipoprotein C3
viltolarsen	Viltepso	NS Pharma	2020	Duchenne muscular dystrophy	ASO	Splicing modulation	Exon 53 of *DMD*
casimersen	Amondys 45	Sarepta Therapeutics	2021	Duchenne muscular dystrophy	ASO	Splicing modulation	Exon 45 of *DMD*

^1^ Volanesorsen did not receive FDA approval in 2018 but was approved by European Medicines Agency (EMA) in 2019.

**Table 2:** Summary of current oligonucleotide therapeutics in clinical trials for oncology.

Oligonucleotide Therapeutics	Target	Drug Modality	Cancer Types	Clinical Trials
DNAzyme targeting EBV-LMP1 (DZ1)	*EBV-LMP1*	DNAzyme	Nasopharyngeal Cancer	NCT01449942
68Ga-Sgc8	*PTK7*	aptamer	Colorectal Cancer	NCT03385148
EYE001 (Anti-VEGF Pegylated Aptamer)	*VEGF*	aptamer	Retinal Cancer	NCT00056199
NOX-A12	*CXCL12*	aptamer	Pancreatic Cancer|Colorectal Cancer|Myeloma|Leukemia	NCT01521533|NCT01521533|NCT03168139
AS1411	*NCL*	aptamer	Acute Myeloid Leukemia	NCT01034410|NCT00881244|NCT00740441|NCT00512083
KRAS G12D siRNA	*KRASG12D*	siRNA	Pancreatic Cancer	NCT03608631
EphA2-targeting DOPC-encapsulated siRNA	*EPHA2*	siRNA	Solid Tumors	NCT01591356
APN401	*CBLB*	siRNA	Brain Cancer|Melanoma|Pancreatic Cancer|Renal Cell Cancer	NCT03087591|NCT02166255
Proteasome siRNA and tumor antigen RNA-transfected dendritic cells	*LMP2*, *LMP7*, *MECL1*	siRNA	Melanoma	NCT00672542
TKM-080301	*PLK1*	siRNA	Cancer with hepatic metastases| Liver Cancer|Hepatocellular Cancer| Adrenocortical Cancer	NCT01437007|NCT02191878|NCT01262235
Atu027	*PNK3*	siRNA	Solid Tumors|Pancreatic Cancer	NCT00938574|NCT01808638
DCR-MYC	*MYC*	siRNA	Solid Tumors|Hepatocellular Cancer	NCT02110563|NCT02314052
CALAA-01	M2 subunit of ribonucleotide reductase (R2)	siRNA	Solid Tumors	NCT00689065
siG12D LODER	*KRASG12D*	siRNA	Pancreatic Cancer	NCT01676259|NCT01188785
ARO-HIF2	*HIF2A*	siRNA	Clear Cell Renal Cell Carcinoma	NCT04169711
SV40 vectors carrying siRNA	Unknown	siRNA	Chronic Myeloid Leukemia	NCT00257647
MRX34	30 unique oncogenes, including but not limited to *MET*, *MYC*, *PDGFRA*, *CDK4/6* and *BCL2*	miRNA	Liver Cancer|Lung Cancer |Lymphoma |Melanoma|Multiple Myeloma|Renal Cell Cancer|	NCT01829971|NCT02862145
INT-1B3	*JNK1*	miRNA	Solid Tumor	NCT04675996
TargomiRs	Multiple oncogenes, including *BCL2*, *MCL1*, *CCND1*, and *WNT3A*	miRNA	Malignant Pleural Mesothelioma|Non-Small Cell Lung Cancer	NCT02369198
Cobomarsen (MRG-106)	*MIR155*	miRNA	Cutaneous T-Cell Lymphoma|Lymphoma|Leukemia	NCT03837457|NCT03713320|NCT02580552
1018 ISS	*TLR9*	ASO	Non-Hodgkin’s Lymphoma|Colorectal Cancer	NCT00251394|NCT00403052
AEG35156	*XIAP*	ASO	Hepatocellular Cancer|Pancreatic Cancer|Breast Cancer|Non-Small Cell Lung Cancer| Leukemia|Lymphoma	NCT00357747|NCT00363974|NCT00372736|NCT00385775|NCT00557596|NCT00558545|NCT00558922|NCT00768339|NCT00882869|NCT01018069
Apatorsen (OGX-427)	*HSP27*	ASO	Urologic Cancer|Bladder Cancer|Prostate Cancer|Urothelial Cancer|Non-Small Cell Lung Cancer	NCT00487786|NCT01454089|NCT01681433|NCT01780545|NCT01829113
ARRx (AZD5312)	*AR*	ASO	Prostate Cancer	NCT02144051|NCT03300505
AZD4785	*KRAS*	ASO	Non-Small Cell Lung Cancer	NCT03101839
AZD8701	*FOXP3*	ASO	Advanced Cancer	NCT04504669
AZD9150	*STAT3*	ASO	Bladder Cancer|Lymphoma|Malignancies	NCT02546661|NCT02549651|NCT03394144|NCT03527147|NCT03819465
BP1001	*GRB2*	ASO	Ph1 Positive Leukemia|Acute Myeloid Leukemia|Chronic Myelogenous Leukemia	NCT01159028|NCT02781883|NCT02923986|NCT04196257
Cenersen (EL625)	*TP53*	ASO	Acute Myelogenous Leukemia|Lymphoma	NCT00074737|NCT00636155|NCT00967512|NCT02243124
CpG 7909 (PF03512676)	*TLR9*	ASO	Melanoma|Breast Cancer|Renal Cancer|Lymphoma|Non-Small Cell Lung Cancer|Esophageal Cancer|Prostate Cancer	NCT00031278|NCT00040950|NCT00043368|NCT00043394|NCT00043407|NCT00043420|NCT00070629|NCT00070642|NCT00085189|NCT00112242|NCT00145145|NCT00185965|NCT00199836|NCT00226993|NCT00233506|NCT00292045|NCT00299728|NCT00369291|NCT00438880|NCT00471471|NCT00490529|NCT00669292|NCT00819806|NCT00824733|NCT00880581|NCT01266603|NCT01588015
CpG ODN (GNKG168)	*TLR9*	ASO	Leukemia	NCT01035216|NCT01743807
CpG Oligonucleotide ^1^	*TLR9*	ASO	Breast Cancer	NCT00640861
CpG-ODN ^1^	*TLR9*	ASO	Glioblastoma	NCT00190424
Custirsen (OGX-011)	*ApoJ*	ASO	Prostate Cancer|Breast Cancer|Non-Small Cell Lung Cancer	NCT00054106|NCT00138658|NCT00138918|NCT00258375|NCT00258388|NCT00327340|NCT00471432|NCT01083615|NCT01188187|NCT01497470|NCT01578655|NCT01630733
Danvatirsen (AZD9150, ISIS STAT3Rx)	*STAT3*	ASO	Advanced Cancers	NCT01563302|NCT01839604|NCT02417753|NCT02417753|NCT02499328|NCT02983578|NCT03334617
EGFR Antisense DNA	*EGFR*	ASO	Head and Neck Squamous Cell Cancer|Gastric Cancer|Ovarian Cancer|Prostate Cancer	NCT00009841|NCT00023634|NCT00903461|NCT01592721|NCT03433027
EZN-2968 (RO7070179,SPC2968)	*HIF1A*	ASO	Hepatocellular Cancer|Lymphoma	NCT00466583|NCT01120288|NCT02564614|NCT00466583
G4460	*CMYB*	ASO	Leukemia| Hematologic Malignancies	NCT00002592| NCT00780052
IGF-1R/AS ODN	*IGF1*	ASO	Glioma	NCT01550523|NCT02507583
IGV-001 containing autologous GBM cells treated with antisense oligonucleotide (IMV-001)	*IGF1R*	ASO	Glioblastoma	NCT04485949
IMO-2055 (EMD 1201081)	*TLR9*	ASO	Renal Cell Cancer|Colorectal Cancer|Non-Small Cell Lung Cancer|Head and Neck Cancer	NCT00729053|NCT01040832|NCT00633529|NCT00719199|NCT01360827
ION251	*IRF4*	ASO	Myeloma	NCT04398485
ION537	*YAP1*	ASO	Advanced Solid Tumors	NCT04659096
ISIS 183750(ISIS-EIF4ERx, LY2275796)	*EIF4E*	ASO	Castrate-Resistant Prostate Cancer|Non-Small Cell Lung Cancer|Colorectal Cancer	NCT00903708|NCT01234025|NCT01234038|NCT01675128
ISIS 2503	*HRAS*	ASO	Colorectal Cancer|Pancreatic Cancer	NCT00004193|NCT00005594|NCT00006467
ISIS 5132	*CRAF*	ASO	Ovarian Cancer	NCT00003892
L-Bcl-2 antisense oligonucleotide	*BCL2*	ASO	Advanced Lymphoid Malignancies	NCT04072458
LErafAON	*CRAF*	ASO	Cancers	NCT00024648|NCT00024661|NCT00100672
Lucanix	*TGFB2*	ASO	Non-small Cell Lung Cancer	NCT01058785|NCT01279798
LY2181308	*BIRC5*	ASO	Non-small Cell Lung Cancer	NCT01107444
LY900003 (ISIS 3521, Affinitak)	*PKCA*	ASO	Melanoma|Lung Cancer|Non-Small Cell Lung Cancer|Breast Cancer	NCT00003989|NCT00017407|NCT00034268|NCT00042679|NCT00042679|NCT00003236
MTL-CEBPA	*CEBPA*	ASO	Hepatocellular Cancer	NCT02716012|NCT04105335|NCT04710641
Oblimersen (G3139)	*BCL2*	ASO	Cancers	NCT00003103|NCT00004862|NCT00004870|NCT00005032|NCT00016263|NCT00017251|NCT00017589|NCT00017602|NCT00021749|NCT00024440|NCT00030641|NCT00039117|NCT00039481|NCT00042978|NCT00047229|NCT00049192|NCT00049374|NCT00054548|NCT00054639|NCT00055822|NCT00059813|NCT00060112|NCT00062244|NCT00063934|NCT00064259|NCT00070083|NCT00070343|NCT00078234|NCT00079131|NCT00080847|NCT00085124|NCT00085228|NCT00086944|NCT00091078|NCT00301795|NCT00409383|NCT00517218|NCT00518895|NCT00542893|NCT00543205|NCT00543231|NCT00636545|NCT00736450|NCT01200342
OGX-427	*HSP27*	ASO	Cancers	NCT00487786|NCT00959868|NCT01120470|NCT01844817|NCT02423590
PNT2258	*BCL2*	ASO	Prostate Cancer|Lymphoma|Melanoma	NCT01191775|NCT01733238|NCT02226965
SPC2996	*BCL2*	ASO	Chronic Lymphocytic Leukemia	NCT00285103
TGFβ2 Antisense-GMCSF Gene Modified Autologous Tumor Cell (TAG) Vaccine	*TGFB2*	ASO	Advanced Cancer	NCT00684294
SD-101	*TLR9*	ASO	Cancers	NCT01042379|NCT01745354|NCT02254772|NCT02266147|NCT02521870|NCT02731742|NCT02927964|NCT03007732|NCT03322384|NCT03410901|NCT04050085|NCT03831295
Trabedersen (AP 12009, OT-101)	*TGFB2*	ASO	Glioblastoma|Anaplastic Astrocytoma|Pancreatic Cancer|Melanoma|Colorectal Cancer	NCT00431561|NCT00761280|NCT00844064
VEGF-Antisense Oligonucleotide	*VEGF*	ASO	Mesothelioma	NCT00668499

^1^ as no additional information is available about these two CpG oligonucleotides on ClinicalTrials.gov accessed on 15 March 2021, we treat them as two different CpG oligonucleotides.

**Table 3:** ASO chemical modifications and pharmacology profiles.

ASO Modification	Modification Type	Nuclease Resistance	RNAse H Cleavage	Target Affinity	Toxicity
Phosphorothioate (PS)	Phosphate	+	Yes	-	++
Phosphoramidate (NP)	Phosphate	+	No	+	+
Methyl-phosphonate (MP)	Phosphate	+	No	-	+
2′-O-methyl (2′-OMe)	Ribose	++	No	++	+ Reduced immune activation; less toxic than PS
2′-O-methoxyethyl (2′-MOE)	Ribose	++	No	++	+ Reduced immune activation; Less than 2′-OMe; less toxic than PS
Phosphorodiamidate morpholino (PMO)	Ribose phosphate	++	No	++	Safer
Locked nucleic acid (LNA)	Ribose	++	No	+++	+++
Constrained ethyl (cEt)	Ribose	+++	No	+++	++
Peptide nucleic acid (PNA)	Ribose phosphate	++	No	++	++ No immune activation

## Data Availability

Not applicable.

## Supplementary Materials

The following are available online at https://www.mdpi.com/1422-0067/22/7/3295/s1. Summary of clinical trials of oligonucleotide therapeutics in oncology as of 01/01/2021.

## Abbreviations

ASO	Antisense Oligonucleotide
PN	Phosphoramidate
PS	Phosphorothioate
MP	Methyl-phosphonate
OS	Overall survival
PFS	Progression-free survival
